# Sentence memory recall in adolescents: Effects of motor enactment, keyboarding, and handwriting during encoding

**DOI:** 10.1002/brb3.3226

**Published:** 2023-08-21

**Authors:** Göran B. W. Söderlund, Silje Torvanger, Nouchine Hadjikhani, Jakob Åsberg Johnels

**Affiliations:** ^1^ Faculty of Teacher Education Arts and Sports Western Norway University of Applied Sciences Sogndal Norway; ^2^ Department of Education and Special Education University of Gothenburg Gothenburg Sweden; ^3^ Gillberg Neuropsychiatry Centre University of Gothenburg, Sahlgrenska Academy Göteborg Sweden; ^4^ Institute of Neuroscience and Physiology Speech and language pathology Unit & the Gillberg Neuropsychiatry Centre, University of Gothenburg Gothenburg Sweden; ^5^ Harvard Medical School Martinos Center for Biomedical Imaging Massachusetts General Hospital Boston Boston Massachusetts USA; ^6^ Trudvang Skule Sogndal Norway

**Keywords:** enactment, encoding, handwriting, keyboard writing, memory recall

## Abstract

**Background:**

Prior research has shown that memory for action sentences is stronger when stimuli are enacted during encoding than simply listened to: the so‐called enactment effect. The goal of the present study was to explore how writing during encoding—through handwriting and through keyboarding—fares compared with enacting, in supporting memory recall.

**Methods:**

One hundred Norwegian high school students (64 girls, 36 boys) aged 16–21 years (M = 17.1) participated in the study. Four lists of verb–noun sentences with 12 sentences in each list were presented in four encoding conditions: (i) motor enactment, (ii) verbal listening, (iii) handwriting, and (iv) keyboarding.

**Results:**

Results revealed a significant main effect of encoding condition, with the best memory gained in the enactment condition. Regarding writing, results showed that handwriting and keyboarding during encoding produced the lowest recall in comparison with the enactment and verbal listening conditions.

**Conclusion:**

These results thus provide additional support for the enactment effect. While there has been much discussion on the relative benefits of handwriting versus keyboarding on student performance, both seemed to be equally poor strategies for the particular learning task explored here, potentially through increased cognitive load.

## Graphical abstract

We compared recall of verb–noun sentences under four encoding conditions: handwriting, keyboard writing, listening, and enacting. Based on prior research showing a robust effect of enactment, our goal was to explore how handwriting and keyboard writing fares in comparison with enacting. One hundred high school students participated. Results revealed that verb–noun sentences are best recalled when enacted, while the poorest recall was found in handwriting and keyboarding that produced the same results.

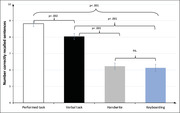

## 1 INTRODUCTION

Does enacting the meaning of action sentences to be remembered result in deeper encoding and better recall than passive listening? And does writing—handwriting or on a computer—during encoding of action sentences—improve memory recall? These two questions are addressed in the present study because of their importance for the learning sciences as well as for the practical efforts of educators.

The field of embodied cognition emphasizes the significance of students’ physical body involvement in thinking and learning (Shapiro & Stolz, 2018). A particularly well‐replicated finding in this context is the so‐called motor enactment effect, or performance effect, defined as the fact that memory recall for action sentences is enhanced if the learner performs a semantically related act during encoding, using gestures or other bodily movements (Cohen, 1989; Nilsson, 2000; Nyberg & Nilsson, 1995). For instance, the likelihood of a person recalling the sentence “Eat with the spoon” is considerably larger if the person performs semantically related movements during encoding. This experimental effect is robust across different ages and populations (Badinlou et al., 2017; Rönnlund et al., 2003). Performed encoding also displays potential educational relevance, for instance, in foreign language acquisition (Porter, 2016), chemistry education (Stull et al., 2018), and in supporting comprehension of instructions among students with special educational needs (Xie et al., 2022).

The experimental evidence of the enactment/performance effect has been demonstrated by comparison with verbal listening (often referred to as verbal task), a condition in which the learner simply listens to sentences during encoding. Two main types of arguments have been put forward to explain the enactment effect in these studies: that the enactment encourages a deeper processing of the material compared with listening and/or that the motoric activity in and of itself promotes encoding (Bäckman et al., 1991; Cohen, 1989). In an effort to probe the underlying cognitive sources of the enactment effect, several studies have included additional control conditions, meant to isolate or account for the enactment effect (Steffens et al., 2015). For instance, von Essen and Nilsson (2003) included an additional control condition in the form of Swedish sign language. In the sign language condition, the subjects were instructed to sign the action sentences back to the experimenter. Interestingly, the results showed increased recall in both the standard motor enactment and the sign language condition compared to the verbal task. This was interpreted as the demonstration that what matters is the fact of being *motorically active* during encoding—regardless of the nature of the specific movements. However, the authors did not consider the fact that the iconicity of (Swedish) sign language is considerable (Östling et al., 2018). Thus, the lack of difference between the sign language and motor enactment conditions on memory recall might have been due to a considerable overlap between the conditions. Indeed, other research has confirmed that making unrelated movements during encoding does *not* enhance memory recall (Sivashankar & Fernandes, 2022; Zimmer & Engelkamp, 2003). Furthermore, a recent study utilizing both behavioral data and event‐related brain potentials suggested that imagery processing of the to‐be‐remembered material seems to be a necessary aspect of the enactment effect (Ma et al., 2021).

In the current study, we explored the enactment/performance effect during encoding in senior high school students, by contrasting its influence on memory recall not only to encoding through listening (verbal task), but also to encoding through the act of writing (by hand and on a keyboard). In doing so, we moved beyond prior investigations, contrasting the enactment/performance effect with a more commonly employed learning strategy of writing for improved recall. The only similar comparison we are aware of is that by Sivashankar and Fernandes (2022; experiment 2). However, in that study, writing was explored by having participants make gestures of letters in the air, which is clearly very different from an ordinary writing situation. Besides being an educationally relevant comparison, writing involves, much like enactment, a motor component, as well as the need to thoroughly process the to‐be‐remembered (TBR) material, making the comparison also theoretically interesting. The motor activity as such during encoding has since long been suggested to provide an independent contribution to improved memory recall due to multimodal encoding (Bäckman, 1985). Furthermore, prior research on the role of writing (including notetaking) during learning activities has resulted in mixed findings. While the external storage potential of producing and reviewing notes is clearly beneficial for learning (Jansen et al., 2017), the question of whether writing as such supports improved memory recall through stronger encoding is less clear. On the one hand, there is evidence in favor of the commonly held view that the act of writing entails the learner to be alert, focused, and active, which in and of itself might support deepened encoding compared to listening (Bui & Myerson, 2014; Mangen & Velay, 2010; Mangen et al., 2015). Moreover, in research on vocabulary learning, a so‐called orthography effect has been observed (Ricketts et al., 2009), meaning that the presence of a word's written form boosts learning of that vocabulary item (Ricketts et al., 2009). On the other hand, writing during encoding has also been shown to increase the cognitive load (Sweller, 2011) and lead to poorer learning outcomes, at least for certain types of learning material, for certain learners and for certain assessment formats (Jansen et al., 2017). Consequently, whether the activity of writing during encoding of action sentences supports or hinders memory recall in adolescents is a topic in need of more empirical investigation.

Another novel feature of the present study is that the writing control activity was experimentally subdivided into two conditions, one for handwriting and one for keyboarding/typing. There are some controversies on the relative benefits of the mode of writing activity on learning and memory. Handwriting includes both visual perception and motor action, that is, haptics, defined as the combination of tactile perception with active movements, while typewriting is divided into two distinct, and spatiotemporally separated, spaces: the motor space (the keyboard) and the visual space (the screen) (Mangen & Velay, 2010). One might, perhaps, hypothesize that the haptic component in handwriting might yield a similar effect for successful encoding as of enactment. Indeed, since long an interaction between motor‐ and visual memory in graphic form memory has also been shown (Hulme, 1979). Furthermore, in direct comparisons between the role of typing and handwriting for developing letter recognition, handwriting produced better results in children (Longcamp et al., 2005) and adults (Longcamp et al., 2008). In addition, an influential study of college students learning from lectures showed that taking notes by hand stood out as superior to keyboarding (Mueller & Oppenheimer, 2014). However, this conclusion has not been well replicated (Morehead et al., 2019), and critical questions remain unanswered regarding the possible superiority of one writing mode over another.

To sum up, we have known since long that memory for action sentences is more stable and robust when they are enacted/performed during encoding rather than simply listened to—the so‐called enactment effect. The goal of the present study was to explore how writing during encoding—through handwriting or through keyboarding—fares in comparison to the encoding strategy when it comes to strengthening memory recall.

## 2 METHODS

### 2.1 Participants

One hundred children (64 girls, 36 boys) aged 16–21 years (*M* = 17.1) participated in the study. All participants were pupils from higher education preparatory programs of upper secondary schools in western Norway. This selection of participants was meant to guarantee that they were experienced practitioners of both handwriting and keyboarding. Data on self‐estimated use of keyboarding and handwriting per week were also collected. There are two officially recognized written varieties of Norwegian, Nynorsk and Bokmål. Bokmål, which has the highest number of users (85%−90%), derives from the 19th‐century Danish, whereas Nynorsk represents a re‐establishing of Norwegian as a written language based on the rural Norwegian dialects of the 19th century. However, for the last hundred years, the two written languages have followed the same orthographic principles for letter‐sound correspondences (i.e., grapheme‐phoneme mapping) (Vangsnes et al., 2017). To ensure that the variety of written languages did not influence the results, we made a comparison of the participants by language group. No effect of written language was found, neither main effect nor in any of the four encoding conditions tested separately, and these data are therefore not reported further. The participants received the test in their preferred written code. Participant's grades in both written alternatives of Norwegian were also collected, and all participants reached at least a passing grade in both forms (grade 3 or above on a six‐graded scale). For participant characteristics, see Table 1.

**TABLE 1:** Participant characteristics.

	Test language		Age	Mean grade in Norwegiana^a^			Handwrite^b^	Laptop write^b^
Participants	NN	BM	*M* (SD)	NN	BM	Oral	Min/week	Min/week
Boys (N)	26	10	17.0 (0.68)	3.8	4.0	4.4	49.3	96.7
Girls (N)	21	43	17.2 (0.78)	4.1	4.3	4.9	45.6	109.9
Total (N)	47	53	17.1 (0.75)	4.0	4.2	4.7	47.0	105.2

Abbreviations: BM, Bokmål; NN, Nynorsk.

^a^ Grades in Norwegian (1–6), no participants had the lowest grades (1, 2).

^b^ Self‐estimated use per week.

### 2.2 Ethics

Written informed consent was obtained from all participants before starting the testing. Participants were also informed that they could withdraw from the study at any timepoint without giving any reason. This study was conducted after approval from Norwegian Centre for Research Data (NSD 2017/52910).

### 2.3 Design

An experimental within‐subjects (1 × 4) design was used in which memory recall was compared following each of the four encoding conditions: (i) motor enactment (performed) task, (ii) verbal listening, (iii) handwriting, and (iv) keyboarding.

### 2.4 Materials

The TBR items consisted of 48 verb–noun action sentences divided into four separate lists with 12 sentences in each list, and with one list for each encoding condition. Each sentence consisted of a unique verb and a unique noun (e.g., “roll the ball”). The sentences were placed in random order. All TBR sentences were presented verbally from a laptop with loudspeakers.

### 2.5 Procedure

Participants were tested individually. The experiment lasted for about 30 min, including instructions. First, two training sentences were presented. The four encoding conditions were counterbalanced across participants, so that each list of 12 action sentences (e.g., roll the ball; write with the pen) in each condition could present the same number of times in every encoding condition. List‐order (1−4) and encoding condition‐order (1–4) were also counterbalanced. Every verb–noun sentence was played out loud from a laptop with an interval of 10 s for each TBR sentence and 3 s in between sentences to provide enough time to write or act the sentences. This made an inter‐stimulus‐interval (ISI) of totally 13 s , in all 2 min 30 s for each list. In order to be able to compare results to previous research (e.g., Bäckman et al., 1991; Nilsson, 2000; Rönnlund et al., 2003), the present protocol followed procedures used in earlier studies of motor performed tasks, with direct free recall after each presented sentence list. However, one adjustment from earlier protocols was made, where we increased the ISI times from 5 s to 13 s in order to provide participants enough time to write down the sentences in the two writing conditions (handwrite and keyboarding). Participants were instructed to recall as many sentences as possible after each list, in any order. In the handwriting and keyboarding conditions, they were instructed to write down each sentence heard in verbatim. The ISI of 13 s provided enough time to write the sentences without any need to hurry. Laptop, paper and pencil, and objects for enactment were provided during the encoding phase and removed and hidden during the recall phase. Directly after the presentation of the last item in a list, participants performed a free‐recall test in which they spoke out loud as many sentences as possible, in any order. Maximum time for memory recall was 2 min, which was more than enough, and no participants exceeded the recall time or used more than 1 min for recall. The experiment lasted for approximately 30 min. Consistent with earlier subject performed task (SPT) studies, strict scoring was used for the nouns (exact matches were required), and lenient scoring was used for the verbs (exact matches not required) (e.g. Söderlund et al., 2007). That is, correct noun was sufficient for a correct answer.

### 2.6 Statistical analysis

We used a repeated measures analysis of variance (ANOVA), a 4 × 1 design, to assess the main effect of encoding. As post hoc tests, we used paired samples *t*‐test where we compared the six possible pairs of encoding conditions to determine if the differences were significant. We made a Bonferroni correction for multiple comparisons (six comparisons). This corresponds to an adjusted *p*‐value for significance of *p* < .0083.

## 3 RESULTS

The repeated measures ANOVA, with encoding condition (4 × 1) as the within‐individual factor, showed a significant effect of encoding condition, *F*(3,97) = 60.12, *p* < .001, *η* ^2^ = 0.650. The encoding condition resulting in the highest number of recalled items was enactment (*M* = 8.8) followed by the verbal task condition (i.e., listening; *M* = 8.0). The poorest performance was found in the handwriting (*M* = 6.2) and keyboarding (*M* = 6.1) conditions.

**FIGURE 1:**
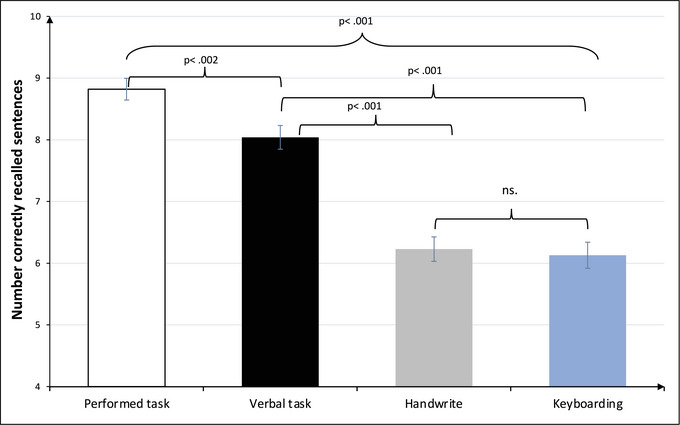
Number of correctly recalled verb–noun sentences as a function of encoding condition. *Note*: Error bars represent standard error of the mean.

Post hoc testing, using paired samples *t*‐tests between the four encoding conditions, confirmed that the difference between enacted task and the other three conditions were significant: (i) verbal task (*t*(99) = 3.22, *p* = .002), (ii) keyboarding (*t*(99) = 10.93, *p* < .001), and (iii) handwriting (*t*(99) = 10.75, *p* < .001. Performance in the verbal task condition was higher compared to keyboarding (*t*(99) = 8.07, *p* < .001] and handwriting conditions (*t*(99) = 8.49, *p* < .001]. The last post hoc comparison showed no difference between the handwriting and keyboarding conditions, *t*(99) = .45, *p* = .652. See Figure 1 for a visual presentation of the results and Table S1 for exact figures and effect sizes.

Two more sensitivity analyses were conducted to control for possible confounding effects. First, we conducted a repeated measures ANOVA with list order (4 × 1) across encoding conditions as the within‐individual factor. There was no evidence that performance changed depending on list order (*F*(3,297) = .371, *p* = .774). Second, we controlled whether the strictness of the scoring affected the results (both correct verb and noun required). The overall score became slightly lower with strict scoring (both verb and noun correct, *M* = 6.7) compared to lenient scoring (noun correct, 7.3). Importantly, the differences between the four encoding conditions remained significant, and effect sizes were almost exactly the same.

## 4 DISCUSSION

The current study demonstrates that motor enactment during sentence encoding clearly benefits memory recall in adolescents. This replicates an effect previously demonstrated in individuals of varying ages (Bäckman & Nilsson, 1985; Badinlou et al., 2017; Rönnlund et al., 2003), and with typical or atypical development and functioning (Söderlund et al., 2007). For the first time, by directly comparing the effects of motor enactment and verbal (listening) strategies with those of handwriting and keyboarding, we also investigated how writing during encoding affected memory recall, in comparison with motor enacting. Somewhat surprisingly, writing, regardless of the mode, seemed to negatively impact memory encoding/recall of action sentences compared with the other two conditions. Thus, our results directly contrast with the idea that writing (handwriting or keyboarding) during a verbal task makes the learner more deeply engaged with the content and facilitates memory encoding when compared with “plain” listening. Furthermore, we show that there is no difference between handwriting and keyboarding on recall performance. There has been much discussion on the relative benefits of handwriting versus keyboarding on student performance (Mangen & Velay, 2010; Mangen et al., 2015; Mueller & Oppenheimer, 2014), but for the particular learning task explored here, namely verbatim action sentence encoding, both seemed to be equally poor strategies. Our results suggest that writing during encoding in a sentence recall task indeed lowered recall performance, potentially through increased cognitive load and thus competition of processing resources that is a prerequisite for successful memory encoding (Cohen, 1989; Sweller, 2011). An increased cognitive load can take up working memory capacity and thus lower performance (Waterman et al., 2017) and the motor encoding as such can offload working memory during encoding and thus produce better memory performance (Allen & Waterman, 2015).

It is important to highlight that in this short report, we only focused on sentence memory recall in an experimental setting. Thus, in order to move the knowledge forward, future research is needed to explore how these strategies relate to a broader set of learning contents and outcomes. Here, a potentially important distinction needs to be made between notetaking during academic work, and verbatim memory recall of unrelated verb‐noun sentences. These activities occur on different processing levels, and notetaking during lectures is more elaborate in comparison with verbatim recall. Notetaking versus verbatim writing can be regarded as deep versus shallow encoding, referring back to the classical *levels of processing model* proposed by Craik and Lockhart (1972). That being said, our results clearly indicate that empirical testing is indeed needed to capitalize the effects of enactment for more natural learning content, and more generally to probe the truth‐value of general claims of the beneficial effect of writing during learning for different populations.

The results of this study inform theoretical interpretations of the enactment/performance effect. Specifically, enactment and writing involve some shared aspects, including a motor component as well as the need to focus and to process the information. Our novel comparison thus suggests that since enactment, but not writing, supported memory recall for action sentences, it is less likely that these common mechanisms are responsible for the enactment effect. Instead, our results further justify the search for alternative mechanisms, such as the fact that enactment might require us to *imagine* the to‐be‐remembered event in the action sentence, which may be critical for more robust/deeper memory encoding (Ma et al., 2021).

## 5 LIMITATIONS AND CONCLUSIONS

The task performed by the subjects is an episodic memory paradigm, where working memory also plays a role. If the participants first recall the last sentences they heard, these will be stored in working memory. The rest of the sentences will be a part of long‐term memory, but in short term storage (Craik & Lockhart, 1972; Craik & Watkins, 1973). It would have been interesting to explore if these differences in recall performance would remain the same after memory consolidation into long‐term storage, preferably after a night's sleep. Sleep benefits are essential in declarative memory recall (Diekelmann et al., 2009). Another related limitation is that no information was gathered from our participants with regard to factors that could potentially affect individual memory performance, such as exercise, medication, or caffeine intake during the day of testing. However, given the within‐subject design of this study, we estimate that these could only play a minor role in our results. Nevertheless, we encourage future research to gather more information on factors that could influence this task.

To conclude, the current study demonstrates that motor enactment during action sentence encoding clearly benefits memory recall in adolescents. These results thus provide additional support for the enactment effect. While there has been much discussion of the relative benefits of handwriting versus keyboarding on student performance, both seemed to be equally poor strategies for the particular learning task explored here, potentially through increased cognitive load.

## AUTHOR CONTRIBUTIONS

GS and ST designed the experiment ST performed the experiment and wrote the first draft in Norwegian. GS analysed data and made tables and the figure GS, JÅJ, and NH wrote and revised the manuscript and cover letters.

## CONFLICT OF INTEREST STATEMENT

The authors declare no conflict of interest.

### PEER REVIEW

The peer review history for this article is available at https://publons.com/publon/10.1002/brb3.3226.

## Supporting information



Table S1. Paired samples t‐test, post hoc comparisons, between all encoding conditions and effects sizes.

## Data Availability

Data can be made available on request from the corresponding author.
