# Supplementary material for: Sentence memory recall in adolescents: Effects of motor enactment, keyboarding, and handwriting during encoding
Source: Brain Behav. 2023 Aug 21;13(11):e3226. doi: 10.1002/brb3.3226 (PMC10636390; doi:10.1002/brb3.3226)
Supplement: Supplementary file 1 — Table S1. Paired samples t‐test, post hoc comparisons, between all encoding conditions and effects sizes. [file BRB3-13-e3226-s001.docx]

**Supplementary material**

Table S1. Paired samples t-test, post hoc comparisons, between all encoding conditions and effects sizes.

| Comparisons of  encoding conditions | Mean (SD) | t-statistics | p-values  (two-sided test) | Effect sizes  Cohen’s d |
| --- | --- | --- | --- | --- |
| Performed task vs. Verbal task | 8.8 (1.8) vs. 8.0 (1.9) | t(99) = 3.22 | .002 | .322 |
| Performed task vs. Handwrite | 8.8 (1.8) vs. 6,2 (2.0) | t(99) = 10.75 | < .001 | 1.075 |
| Performed task vs. Keyboard | 8.8 (1.8) vs. 6.1 (2.1) | t(99) = 10.93 | < .001 | 1.067 |
| Verbal task vs. Handwrite | 8.0 (1.9) vs. 6,2 (2.0) | t(99) = 8.49 | < .001 | .850 |
| Verbal task vs. Keyboard | 8.0 (1.9) vs. 6,1 (2.1) | t(99) = 8.07 | < .001 | .807 |
| Handwrite vs. Keyboard | 6,2 (2.0) vs. 6,1 (2.1) | t(99) = .452 | .652 | .045 |

*Note:* Bonferroni corrections for multiple comparisons made the criterion for a significant p-value <.0083
